# Thermoresponsive Poly(ß-hydroxyl amine)s: Synthesis of a New Stimuli Responsive Amphiphilic Homopolymer Family through Amine-Epoxy ‘Click’ Polymerization

**DOI:** 10.3390/polym11121941

**Published:** 2019-11-25

**Authors:** Jeonghui Hong, Anzar Khan

**Affiliations:** Department of Chemical and Biological Engineering, Korea University, Seoul 02841, Korea; ghdwjd47@korea.ac.kr

**Keywords:** amine-epoxy reaction, ‘click’ chemistry, poly(ß-hydroxyl amine)s, thermoresponsive polymers, amphiphilic homopolymers, ‘click’ polymerization, stimuli responsive polymers

## Abstract

A new synthesis of amphiphilic homopolymers is described. In this synthesis, commercially available and inexpensive primary amines and di-epoxide molecules are utilized as AA- and BB-types of monomers in an amine-epoxy ‘click’ polymerization process. This process can be carried out in water and at room temperature. It does not require a catalyst or inert conditions and forms no byproducts. Therefore, the polymer synthesis can be carried out in open-air and bench-top conditions and a post-synthesis purification step is not required. The modularity of the synthesis, on the other hand, allows for facile structural modulation and tuning of the thermally triggered aggregation process in the temperature range of 7 to 91 °C. Finally, the underlying principles can be translated from linear architectures to polymer networks (hydrogels).

## 1. Introduction

The development of simple synthetic protocols that allow practical access to functional polymers has been a significant goal in polymer chemistry [1]. This notion resonates in the “click” chemistry philosophy [2] especially in the context of material synthesis that emphasizes on, among other things, simplicity of the involved processes [3,4,5,6,7,8,9,10,11]. The idea is that if a synthetic route towards useful materials relies on simple techniques, it can be available to non-experts and as a consequence can have a wider appeal and scope. One class of commercially successful materials that do find such a broad applicability, from chemistry laboratories to our homes, is the epoxy resins. The chemistry here is so simple that by following simple instructions a non-expert [12] can prepare a crosslinked polymer network. In this process, each primary amine reacts twice (NH_2_ = AA monomer) to the glycidyl units. Molecules with multiple amines and epoxides therefore constitute the epoxy network formulations [13].

The amine-epoxy reaction was indeed recognized as one of the potential reactions in the seminal ‘click’ chemistry article by Sharpless and coworkers [2]. It is efficient due to the ‘spring-loaded’ nature of the three-membered epoxy ring [2,8,9,10,11,12,13,14,15,16,17,18,19,20,21,22,23,24,25,26,27,28,29,30,31,32,33,34,35,36,37,38,39]. It does not require a catalyst due to the inherent nucleophilicity of alkyl amines. It does not produce a byproduct. It is not affected by oxygen or moisture. In fact, Sharpless and coworkers pointed out that water could satisfy the demanding range of hydrogen bonding situations arising during an epoxy ring opening reaction. This feature is attractive because water has a high heat capacity and therefore large-scale operations especially involving exothermic polymerization processes can be carried out without requiring an elaborate external mechanism for heat dissipation. Water is also environmentally benign and inexpensive. Overall, therefore, a simple mixing of monomers in water under open-air conditions is enough to set up the polymerization reaction at a targeted scale. In addition, upon full monomer conversion, a post-polymerization purification is also not necessary due to absence of byproducts in the system.

In view of this, in this study, we have employed the amine-epoxy “click” reaction for the synthesis of thermoresponsive polymers. Traditionally, thermoresponsive polymers have been prepared either via anionic or free radical polymerization protocols [40,41,42,43,44,45,46,47]. Both of these technique requires careful handling of the ingredients involved and use of an inert atmosphere. In some cases, careful removal of the polymerization catalysts and ligand is also a necessity. Step-growth polymerizations, in contrast, have not found general utility in the synthesis of thermoresponsive polymers. It is of course noted that living and controlled polymerization protocols are superior in terms of narrow chain length distribution, control over polymer chain-ends, and opportunity to prepare block-type structures as illustrated in works from the laboratories of Lutz [48,49,50,51] and Sumerlin [52,53,54,55,56]. However, a catalyst, heat, purification-free scalable synthesis in air and moisture from a large variety of commercially available and inexpensive building blocks in water is also of considerable significance. These features would allow researchers with no background and infrastructure in polymer synthesis, such as a biologist or an engineer, to access thermoresponsive materials with precise and adaptable chemical structures and adjustable stimuli responsive properties [57,58,59].

Besides practicality of the synthesis, the final molecular structures presented in this work represent a new family of amphiphilic homopolymers [60,61,62,63]. Polymers in which relatively low molecular weight hydrophobic and hydrophilic moieties are present at every repeating unit to attain amphiphilicity. Such polymers are different from amphiphilic block copolymers in which high molecular weight hydrophilic and hydrophobic polymeric segments are connected together to attain amphiphilicity. This small but growing polymer class represents a new molecular design in the preparation of responsive materials.

## 2. Results and Discussion

### 2.1. Linear Polymer Synthesis

**Scheme 1:**
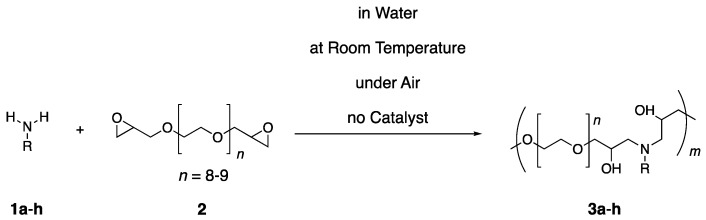
Chemical structures of monomers **1** and **2** and polymer **3** (R: **a** = Me, **b** = Et, **c** = Pr, **d** = Bu, **e** = Pen, **f** = Hex, **g** = Et-OMe, **h** = Et-OEt).

To examine the potential of ‘click’ polymerization between amine and epoxide groups for the synthesis of thermoresponsive polymers, initially, we decided to use amines **1a**–**f** and polyethylene oxide (PEO)-based diglycidyl ether **2** (*M*_n_ = 500 g/mol, *n* = 8–9) as AA and BB-types of monomers, respectively (Scheme 1). It is noteworthy that all of the monomers are commercially available in large quantities (≥0.5 L) and inexpensive.

**Figure 1:**
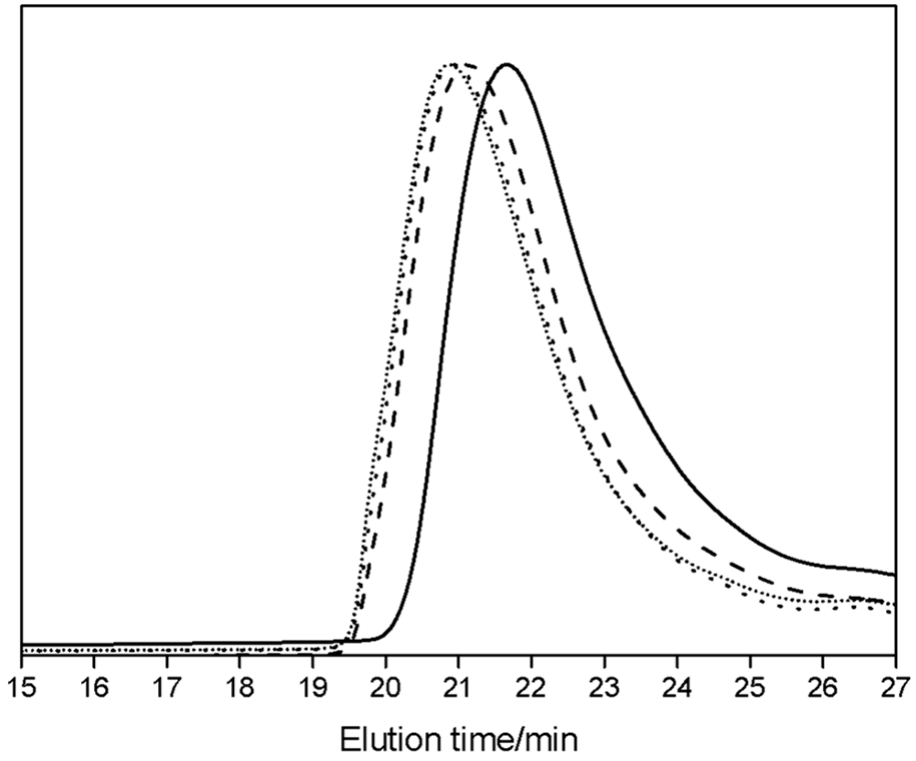
Crude gel permeation chromatography traces for polymer **3d** in tetrahydrofuran as a function of polymerization time (solid line = 12 hr, dash line = 24 hr, small dash line = 36 hr, dot line = 48 hr).

The polymerization reaction involved mixing of monomers and stirring in water. For relatively small scales (a few grams) a screw-cap glass vial was sufficient with magnetic stirring (Figure S1). A > 500-gram synthesis, due to the large scale and a highly viscous nature of the concentrated reaction mixture, requires use of a mechanical stirrer (Figure S2). In both cases, a bench-top operation under ambient conditions was sufficient for a successful polymerization. A crude gel permeation chromatography (GPC) analyses indicated that a 50 wt % aqueous solution resulted into multimodal traces (Figure S3) whereas an 80 wt % solution showed monomodal distribution (Figure 1). Furthermore, the change in molecular weight was negligible after 36 h of polymerization time. Therefore, all polymerizations were carried out for a maximum of 48 h in 80 wt % aqueous solutions. The polymers (**3a**–**f**) were produced in quantitative yields with number average molecular weights ranging from 3–6 kDa against polystyrene standards. The polydispersity index (PDI = *M*_w_/*M*_n_) ranged from 1.2 to 1.5. Such relatively low molecular weight is typical of an AA/BB-type of step-growth polymerization approach since the commercial monomers are used without further purification and one of the monomers (**2**) is already polymeric in nature and polydisperse. These aspects makes it difficult to attain a 1:1 functional group stoichiometry that is critical for high molecular weight polymer formation as predicted by Carother’s equation [64,65]. The practicality aspect of an AA/BB polymerization, however, is of essence to this work. Therefore, alternatives such as synthesis of an AB monomer that would alleviate the stoichiometry imbalance issue were not pursued.

The amine-epoxy polymerization is a fusion process between the active groups of the monomers in water. It is also catalyst-free. Therefore, the system generates purely a high viscosity aqueous solution of polymer chains. No purification step is required if such an aqueous solution can be used. For example, in the thermoresponse studies, one need only add more water to arrive at a dilute aqueous solution typically required for such investigations. In the hydrogel formation, water can be part of the system after the hydrogel synthesis. However, if water needs to be removed, for example for analyses in organic solvents, then a simple freeze-drying process affords neat polymers as colorless viscous materials.

### 2.2. Absence of Over-Alkylation and Branching in the System

**Figure 2:**
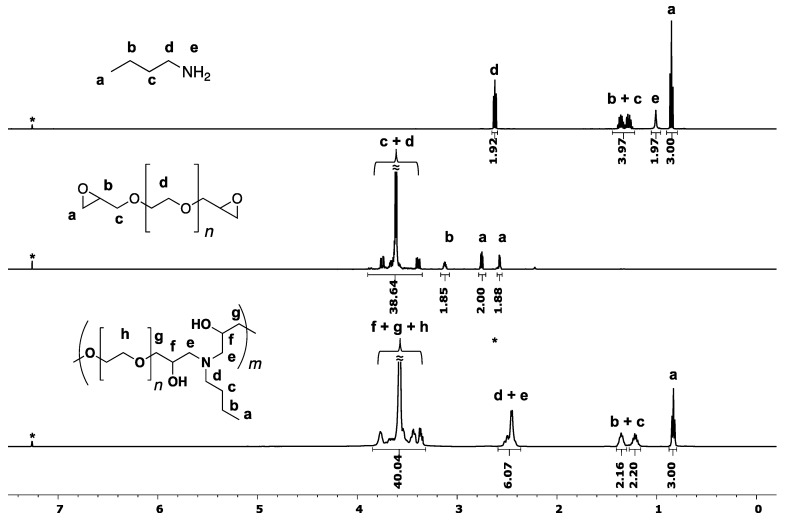
^1^H-NMR traces for monomers **1d**, **2**, and polymer **3d** in deuterated chloroform. Residual solvent signal is shown with an asterisk.

**Figure 3:**
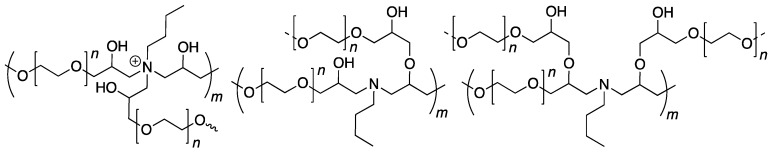
Chemical structures of the possible defects through over-alkylation at the nitrogen (left) and oxygen atoms (middle and right).

**Figure 4:**
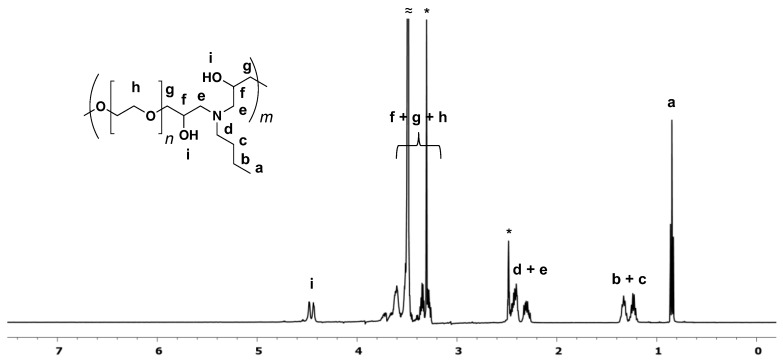
^1^H-NMR of polymer **3d** in deuterated dimethyl sulfoxide. Residual solvent signals are shown with an asterisk.

Area integration analysis in ^1^H-NMR spectroscopy was used to examine the molecular structure in regard to over alkylation at the nitrogen atom or alkylation through alkoxide anion (Figure 2 and Figures S4–S11). To achieve this, the signal from the side-chain methyl group was calibrated to three protons in each spectrum. In comparison, the backbone protons resonating in the range of 3.2–3.8 amounted to be about 40 in each polymer. Among this, 38 belonged to the methylene groups of the ethylene oxide segment and 2 belonged to the protons located at the tertiary carbon atom carrying the hydroxyl group. In the epoxide monomer, these tertiary carbon protons resonated up-field by about 0.6 ppm and were separated from the methylene groups of the ethylene oxide segment, which amounted to be about 38. This meant that for each nitrogen atom, one ethylene oxide chain (from monomer **2**) was incorporated into the repeating unit structure. The newly formed N–CH_2_ protons overlapped with the methylene group of the side-chain and together amounted to be about 6. Over alkylation on the nitrogen atom (Figure 3) would have resulted in the incorporation of one more methylene group adjacent to the nitrogen atom and the integration for N–CH_2_ would become 8. However, this was not the case. The NMR solvent was then changed to deuterated DMSO, in which the hydroxyl protons could be located at 4.5 ppm as a single signal (Figure 4). They amounted to be about 2H per polymer repeat unit and appeared as a doublet due to the coupling with the single proton located at the neighboring tertiary carbon atom. This suggested that the alkoxide anion was indeed quenched and did not participate in the ring opening reaction. If the alkoxides would participate in the polymerization then we would have expected the signal at 3.2–3.8 to represent nearly 80 protons per anion per polymer repeat unit. If both of the possible side reactions would be operative, we would be expecting even a higher degree of mismatch between the side-chain methyl group and the polymer backbone. To observe that per polymer repeating unit, two hydroxyl protons were present along with six N–CH_2_, 2 CH–OH, and 38 ethylene oxide protons suggested absence of the competing side reactions and branching of the polymer structure.

**Figure 5:**
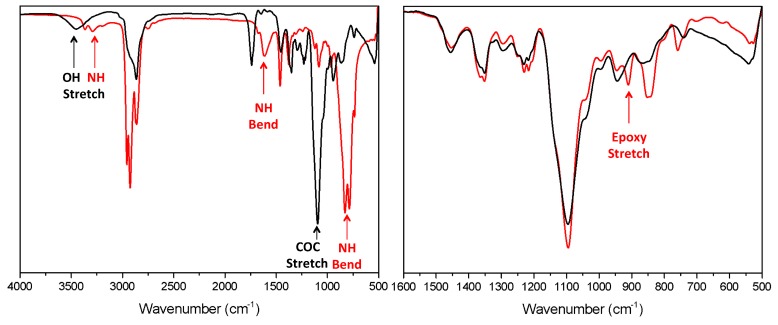
IR spectra of monomer **1d** (red) and polymer **3d** (black; left). The right shows the region of 500–1600 cm^−1^ for monomer **2** (red) and polymer **3d** (black; the full spectra are given in Figure S12).

The hydroxyl groups generated through the amine-epoxy “click” reaction can also be seen in the IR spectrum as a broad signal at 3500 cm^−1^ (Figure 5). The ether C–O stretch from the ethylene oxide segments can be observed as an intense signal at 1100 cm^−1^. The primary N–H stretching and bending vibrations that are sharp intense bands in the region of 3200–3400, 1700, and 600–800 cm^−1^, respectively, cannot be observed in the case of polymers. The asymmetric stretching vibration from C–O of the epoxide group at 911 cm^−1^ was also absent from the spectrum of the polymer (Figure S12). This meant that polymer chains were long enough so that the end groups could not be located either by NMR or IR spectroscopies. These studies also suggest that the polymers were well defined and a repeating unit structure could be assigned to the synthesized materials.

### 2.3. Thermoresponsive Behavior

In thermoresponsive polymers exhibiting a lower critical solution temperature, the polymer chains are initially completely dissolved in water. However, if the temperature of the aqueous polymer solution is raised, the hydrogen bonding interactions between the polymer and the water molecules weakens. This results in the precipitation of dehydrated polymer chains. The entropy-driven phase change is often referred to as a ‘coil to globule transition’ [40,41,42,43,44,45,46,47]. The coil refers to fully solvated (hydrated) polymer chains in a completely dissolved state in water. The globule refers to the non-solvated aggregated state. Since this phase transition depends upon interaction of water molecules with the polymer chain, it can be tuned through incorporation of hydrophobic segments into the polymer chain. In general, as the hydrophobic fraction increases, the hydrogen bonding ability of the polymer chain decreases. Therefore, attractive interactions between the polymer chain and water molecules can be weakened at a lower temperature and the polymer chain aggregation is facilitated through enhanced interaction between the hydrophobic segments.

In the current set of polymers, the hydrophobic segment is a very small carbon chain (C1–C6) that is sparsely populated on the hydrophilic backbone due to the relatively higher molecular weight of the epoxide co-monomer. Therefore, this new polymer family cannot be considered as graft copolymers but as homopolymers in which each polymer-repeating unit contains the hydrophilic (PEG) and hydrophobic (alkyl chain) moieties [60,61,62,63].

**Figure 6:**
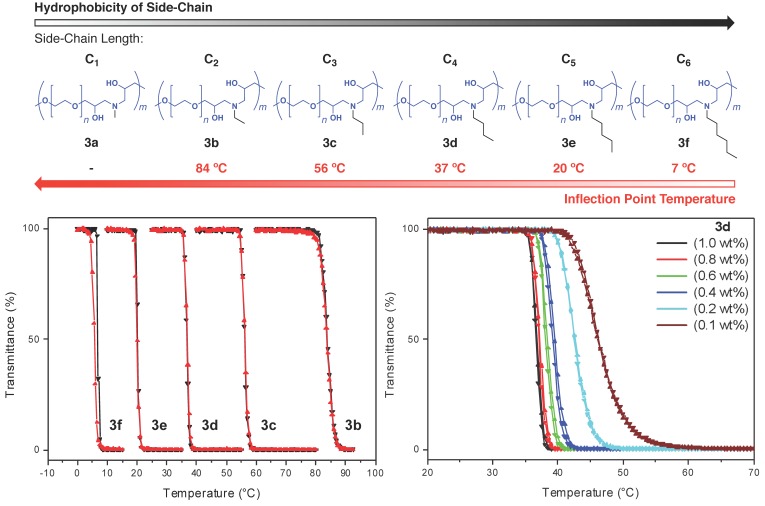
Transmittance at 600 nm as a function of temperature (0.5 °C/min) for aqueous solutions (10 mg/mL) of polymers **3b**–**3f** (left; heating cycles, black triangles; cooling cycles, red triangles). Polymer **3d** at concentrations of 1, 2, 4, 6, 8, and 10 mg/mL (heating cycles, down triangles; cooling cycles, up triangles; right).

Having practical access to the new family of polymers, their assembling behavior under a thermal trigger was then considered. Polymer **3a** having the shortest hydrophobic side chains of methyl groups does not show any phase transition as the polymer chains are mostly composed of hydrophilic ethylene glycol segments which remain soluble in water in the investigated temperature range of 0–100 °C. Raising the hydrophobic content of the polymer chains through increasing the alkyl chain length from C1 (methyl) to C2 (ethyl) allows for observation of the thermal phase transition (defined as the temperature of the inflection point of the heating curve) in polymer 3b at 84 °C (Figure 6). An increase in the alkyl length to C3 (propyl), C4 (butyl), C5 (pentyl), and C6 (hexyl) then allows for achieving a systematic decrease in the phase transition temperature to 56 (**3c**), 37 (**3d**), 20 (**3e**), and 7 °C (**3f**), respectively.

**Figure 7:**
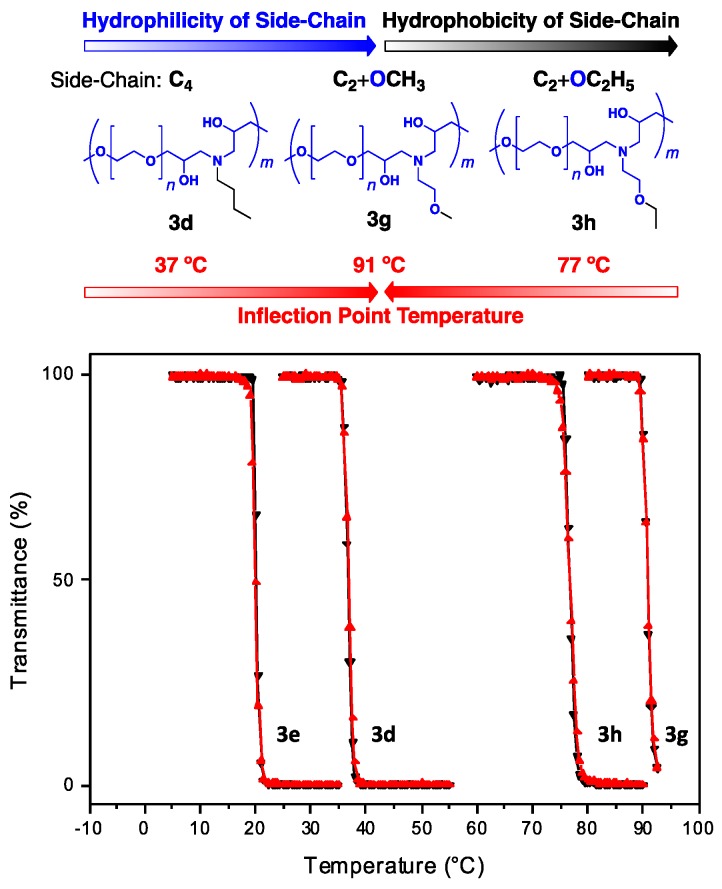
Transmittance at 600 nm as a function of temperature (0.5 °C/min) for aqueous solution (10 mg/mL) of polymers **3d**, **3e**, **3g**, and **3h** (heating cycles, black triangles; cooling cycles, red triangles).

Ready availability of a variety of amine monomers further allows for a change in the hydrophobicity of the polymer side-chain (Figures S10 and S11). For example, addition of an oxygen atom enhances the side-chain capacity to interact with water. Therefore, in comparison to polymer **3d** (37 °C), polymer **3g** shows a much higher thermal transition temperature of 91 °C (Figure 7). A change in the terminal group of methoxy to ethoxy by employing monomer **2h**, allows for increasing the hydrophobicity of the oxygen containing side-chain and as a consequence the transition temperature can be dropped to 77 °C in polymer **3h**.

Another factor that influences the thermal phase transition is the polymer concentration. In general, higher concentration facilitates aggregation. In the case of polymer **3d** for example, the thermal transition point slowly shifts to the higher temperatures and sharpness of the transition decreases upon a decrease in the material concentration (Figure 6).

**Figure 8:**
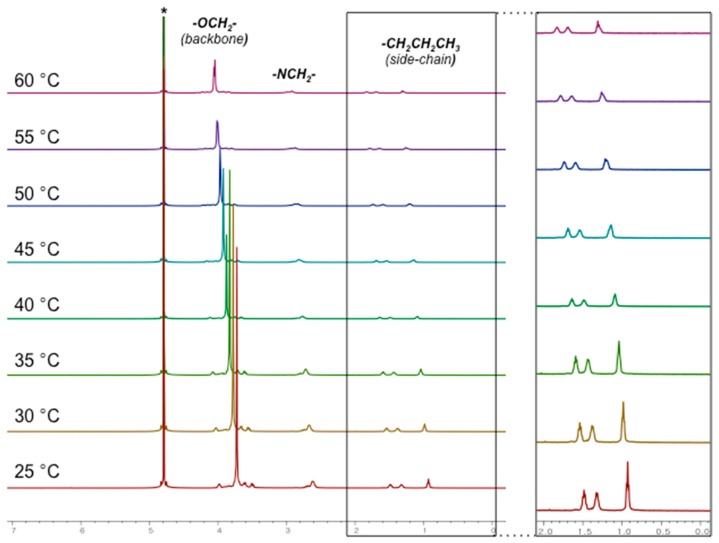
Variable temperature ^1^H-NMR of polymer **3d** in deuterated water (2 mg/mL).

^1^H NMR spectroscopy provides another significant tool in studying the phase separation. For example, the spectrum shows clear and intense proton resonances from the side-chain and backbone in CDCl_3_ indicating free mobility of both segments of the polymer chain (Figure S13). In deuterated water, the resonance from the backbone remains intense but the signal from the alkyl side chain becomes relatively broad and relatively diminished in intensity when compared to the NMR in deuterated chloroform (Figure S13). This indicates that the alkyl side chains have limited segmental mobility and are shielded by the hydrophilic backbone in water. Upon increasing the temperature of aqueous solution, all proton resonances including that of the hydrophilic backbone diminished significantly in intensity, relatively broaden, and shift to higher ppm values (Figure 8). These changes indicate that at higher temperatures, the overall chain mobility decreased and the polymer chains started to collapse on an inter-molecular level.

**Figure 9:**
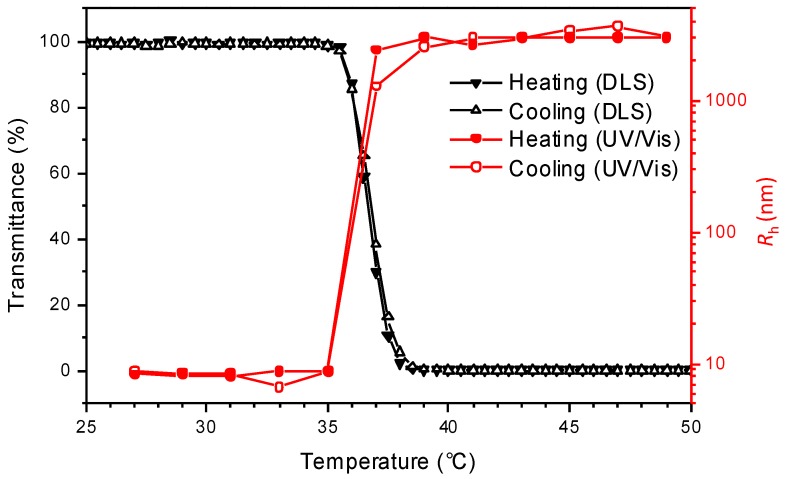
Changes in the hydrodynamic radius (*R*_h_) as a function of temperature as studied by dynamic light scattering (DLS) of an aqueous solution (10 mg/mL) of polymer **3d**. The transmittance data is also shown.

Dynamic light scattering further allows for examining the state of polymer chains in water. As can be seen in Figure 9, below the phase transition temperature, a small particle size was observed. Volume distribution indicated that most of these particles were in the size range of <10 nm. Therefore, the polymer solution appeared clear to the eye and the polymers were largely in a coiled conformation. An increase in the temperature from 35 to 37 °C, however, led to a sudden change in the particle sizes, which now ranged from 1000 to 3000 nm (Figure S14). Such large sizes support the inter-molecular aggregation hypothesis discussed above.

### 2.4. Hydrogel Synthesis

**Figure 10:**
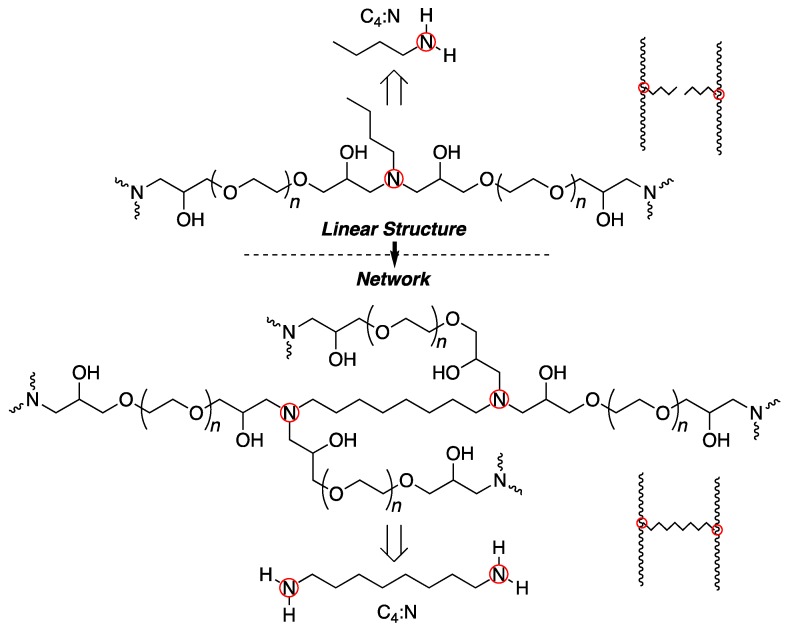
Use of an alkyl diamine as a crosslinker to obtain thermoresponsive hydrogels.

Since a large number of alkyl amines are available commercially, the next step was to examine if thermoresponsive hydrophilic networks could be fabricated. For network formation, a crosslinking point is necessary in the structure. A simple way of achieving this is to use a diamine compound as a monomer. To retain the thermoresponsive character, the spacer between the two amines should be an alkyl chain. Therefore, octyl diamine was used as a crosslinker along with hydrophilic monomer 2 (Figure 10). We hoped that by keeping the hydrophobic to hydrophilic ratio of polymer **3d**, we could render the network sensitive to the biologically relevant temperature range of 37–40 °C. The network formation is carried out under the same conditions that are ideal for synthesis of linear polymers. Due to the presence of polyethylene glycol segments, the network swelled in water. However, since the PEG segments were relatively short, the swelling was relatively low (355%). More importantly, however, when the temperature was increased to 40 °C, the macroscopic samples shrinked and expelled water from inside the networks (Figure S15). The de-swelling was calculated to be about 65%. These results were only preliminary and required further investigations in particular regarding formation of skin layer upon high temperature that did not allow for achieving a 100% de-swelling. Nonetheless, the results indicate that the fundamental concept could be applied to preparation of different polymeric architectures sensitive to temperature.

## 3. Conclusions

To summarize, a large variety of commercially available primary amine and di-epoxide compounds could serve as monomers in amine-epoxy ‘click’ polymerization in water. This process is catalyst-free and can be carried out under air at room temperature. Due to the absence of by-products and catalyst, a post-polymerization purification step is not required. The overall process, therefore, is simple mixing of commercial ingredients in water. If necessary, water can be removed to yield bulk materials. Due to the simplicity, >500-gram scale preparation is possible in a bench-top operation. The produced polymers represent a new family of amphiphilic homopolymers and exhibit thermoresponsiveness. This property is governed by the hydrophilic/hydrophobic balance in the polymer chain and can be modulated through choosing an appropriate amine/epoxide monomer combination. Finally, the concept can be extended to hydrophilic networks through use of a diamine precursor.

## Supplementary Materials

The synthesis and characterization details are available online at https://www.mdpi.com/2073-4360/11/12/1941/s1.

## References

[1] Hawker C.J., Wooley K.L.J.S. (2005). The convergence of synthetic organic and polymer chemistries. Science.

[2] Kolb H.C., Finn M., Sharpless K.B. (2001). Click chemistry: Diverse chemical function from a few good reactions. Angew. Chem. Int. Ed..

[3] Hawker C.J., Fokin V.V., Finn M., Sharpless K.B. (2007). Bringing efficiency to materials synthesis: The philosophy of click chemistry. Aust. J. Chem..

[4] Qin A., Lam J.W., Tang B.Z. (2010). Click polymerization: Progresses, challenges, and opportunities. Macromolecules.

[5] Shi Y., Sun J.Z., Qin A. (2017). Click polymerization: The aurora of polymer synthetic methodology. J. Polym. Sci. A.

[6] Huang D., Liu Y., Qin A., Tang B.Z. (2018). Recent advances in alkyne-based click polymerizations. Polym. Chem..

[7] Li B., Huang D., Qin A., Tang B.Z. (2018). Progress on Catalytic Systems Used in Click Polymerization. Macromol. Rapid Commun..

[8] Lutz J.-F., Börner H.G., Weichenhan K. (2006). Combining ATRP and “click” chemistry: A promising platform toward functional biocompatible polymers and polymer bioconjugates. Macromolecules.

[9] Espeel P., Du Prez F.E. (2014). “Click”-inspired chemistry in macromolecular science: Matching recent progress and user expectations. Macromolecules.

[10] Sumerlin B.S., Vogt A.P. (2010). Macromolecular Engineering through Click Chemistry and Other Efficient Transformations. Macromolecules.

[11] Sanyal A. (2010). Physics, Diels–Alder Cycloaddition-Cycloreversion: A Powerful Combo in Materials Design. Macromol. Chem. Phys..

[12] Members of public are everyday users of the amine-epoxy chemistry commonly referred to as ‘two-part epoxy’ in the market place. For instance, a construction worker uses it to form a protective layer on wooden floors or to cover cracks or to give a glossy finish to a tabletop in the kitchen. Children use its thermosetting nature to fabricate pendants and other ornamental items.

[13] Jin F.-L., Li X., Park S.-J. (2015). Synthesis and application of epoxy resins: A review. J. Ind. Eng. Chem..

[14] Van der Ende A.E., Kravitz E.J., Harth E. (2008). Approach to formation of multifunctional polyester particles in controlled nanoscopic dimensions. J. Am. Chem. Soc..

[15] Van der Ende A., Croce T., Hamilton S., Sathiyakumar V., Harth E. (2009). Tailored polyester nanoparticles: Post-modification with dendritic transporter and targeting units via reductive amination and thiol-ene chemistry. Soft Matter.

[16] Kang T., Amir R.J., Khan A., Ohshimizu K., Hunt J.N., Sivanandan K., Montanez M.I., Malkoch M., Ueda M., Hawker C.J. (2010). Facile access to internally functionalized dendrimers through efficient and orthogonal click reactions. Chem. Commun..

[17] Amir R.J., Albertazzi L., Willis J., Khan A., Kang T., Hawker C.J. (2011). Multifunctional trackable dendritic scaffolds and delivery agents. Angew. Chem. Int. Ed..

[18] Damiron D., Mazzolini J., Cousin F., Boisson C., D’Agosto F., Drockenmuller E. (2012). Poly (ethylene) brushes grafted to silicon substrates. Polym. Chem..

[19] Muzammil E.M., Khan A., Stuparu M.C. (2017). Post-polymerization modification reactions of poly (glycidyl methacrylate) s. RSC Adv..

[20] Ren Y., Jiang X., Yin J. (2009). Poly (ether tert-amine): A novel family of multiresponsive polymer. J. Polym. Sci. A.

[21] Saha A., De S., Stuparu M.C., Khan A. (2012). Facile and general preparation of multifunctional main-chain cationic polymers through application of robust, efficient, and orthogonal click chemistries. J. Am. Chem. Soc..

[22] Si G., Elzes M., Engbersen J., Paulusse J. (2018). Modular Synthesis of Bioreducible Gene Vectors through Polyaddition of N, N′-Dimethylcystamine and Diglycidyl Ethers. Polymers.

[23] Li D., Bu Y., Zhang L., Wang X., Yang Y., Zhuang Y., Yang F., Shen H., Wu D. (2015). Facile construction of pH-and redox-responsive micelles from a biodegradable poly (β-hydroxyl amine) for drug delivery. Biomacromolecules.

[24] Sun G., Liu J., Wang X., Li M., Cui X., Zhang L., Wu D., Tang P. (2019). Fabrication of dual-sensitive poly(β-hydroxyl amine) micelles for controlled drug delivery. Eur. Polym. J..

[25] Su Z., Jiang X. (2016). Multi-stimuli responsive amine-containing polyethers: Novel building blocks for smart assemblies. Polymer.

[26] Liu S., Oderinde O., Hussain I., Yao F., Fu G. (2018). Dual ionic cross-linked double network hydrogel with self-healing, conductive, and force sensitive properties. Polymer.

[27] Yu J., Su Z., Xu H., Ma X., Yin J., Jiang X. (2015). One-pot approach to synthesize hyperbranched poly(thiol–ether amine) (hPtEA) through sequential “thiol–ene” and “epoxy–amine” click reactions. Polym. Chem..

[28] Xu L.Q., Pranantyo D., Neoh K.-G., Kang E.-T., Teo S.L.-M., Fu G.D. (2016). Synthesis of catechol and zwitterion-bifunctionalized poly (ethylene glycol) for the construction of antifouling surfaces. Polym. Chem..

[29] Hamid Z.A., Blencowe A., Ozcelik B., Palmer J.A., Stevens G.W., Abberton K.M., Morrison W.A., Penington A.J., Qiao G.G. (2010). Epoxy-amine synthesised hydrogel scaffolds for soft-tissue engineering. Biomaterials.

[30] Ozcelik B., Brown K.D., Blencowe A., Daniell M., Stevens G.W., Qiao G.G. (2013). Ultrathin chitosan–poly (ethylene glycol) hydrogel films for corneal tissue engineering. Acta Biomater..

[31] Stevens L., Calvert P., Wallace G.G., Panhuis M.I.H. (2013). Ionic-covalent entanglement hydrogels from gellan gum, carrageenan and an epoxy-amine. Soft Matter.

[32] Krüger A.J., Köhler J., Cichosz S., Rose J.C., Gehlen D.B., Haraszti T., Möller M., De Laporte L. (2018). A catalyst-free, temperature controlled gelation system for in-mold fabrication of microgels. Chem. Commun..

[33] Zhou C., Truong V.X., Qu Y., Lithgow T., Fu G., Forsythe J.S. (2016). Antibacterial poly (ethylene glycol) hydrogels from combined epoxy-amine and thiol-ene click reaction. J. Polym. Sci. A.

[34] Liu Z., Zhang C., Xu H., Ma X., Shi Z., Yin J. (2017). A Facile Method Synthesizing Hydrogel Using Hybranched Polyether Amine (hPEA) as Coinitiator and Crosslinker. Macromol. Chem. Phys..

[35] Zhang C., Liu Z., Zhang X., Shi Z., Xu H., Ma X., Yin J., Tian M. (2017). Polyetheramine (PEA): A versatile platform to tailor the properties of hydrogels via H-bonding interactions. Polym. Chem..

[36] Bakarich S.E., Balding P., Gorkin R., Spinks G.M. (2014). Printed ionic-covalent entanglement hydrogels from carrageenan and an epoxy amine. RSC Adv..

[37] Ding J., Zhou C., Li K., Zhang A., Yao F., Xu L., Fu G. (2016). Preparation of well-defined fibrous hydrogels via electrospinning and in situ “click chemistry”. RSC Adv..

[38] Qian S., Zhou C., Xu L., Yao F., Cen L., Fu G. (2014). High strength biocompatible PEG single-network hydrogels. RSC Adv..

[39] Oh J., Jung K.I., Jung H.W., Khan A. (2019). A Modular and Practical Synthesis of Zwitterionic Hydrogels through Sequential Amine-Epoxy “Click” Chemistry and N-Alkylation Reaction. Polymers.

[40] Ward M.A., Georgiou T.K. (2011). Thermoresponsive Polymers for Biomedical Applications. Polymers.

[41] Aseyev V., Tenhu H., Winnik F. (2011). Self organized nanostructures of amphiphilic block copolymers II. Adv. Polym. Sci..

[42] Gibson M.I., O’Reilly R.K. (2013). To aggregate, or not to aggregate? considerations in the design and application of polymeric thermally-responsive nanoparticles. Chem. Soc. Rev..

[43] Roy D., Brooks W.L., Sumerlin B.S. (2013). New directions in thermoresponsive polymers. Chem. Soc. Rev..

[44] Jochum F.D., Theato P. (2013). Temperature-and light-responsive smart polymer materials. Chem. Soc. Rev..

[45] Zhang Q., Weber C., Schubert U.S., Hoogenboom R. (2017). Thermoresponsive polymers with lower critical solution temperature: From fundamental aspects and measuring techniques to recommended turbidimetry conditions. Mater. Horiz..

[46] Atanase L.I., Desbrieres J., Riess G. (2017). Micellization of synthetic and polysaccharides-based graft copolymers in aqueous media. Prog. Polym. Sci..

[47] Atanase L.I., Riess G. (2018). Self-Assembly of Block and Graft Copolymers in Organic Solvents: An Overview of Recent Advances. Polymers.

[48] Lutz J.-F., Hoth A. (2006). Preparation of ideal PEG analogues with a tunable thermosensitivity by controlled radical copolymerization of 2-(2-methoxyethoxy) ethyl methacrylate and oligo (ethylene glycol) methacrylate. Macromolecules.

[49] Lutz J.-F., Akdemir Ö., Hoth A. (2006). Point by point comparison of two thermosensitive polymers exhibiting a similar LCST: Is the age of poly (NIPAM) over?. J. Am. Chem. Soc..

[50] Lutz J.-F., Weichenhan K., Akdemir Ö., Hoth A. (2007). About the phase transitions in aqueous solutions of thermoresponsive copolymers and hydrogels based on 2-(2-methoxyethoxy) ethyl methacrylate and oligo (ethylene glycol) methacrylate. Macromolecules.

[51] Skrabania K., Kristen J., Laschewsky A., Akdemir Ö., Hoth A., Lutz J.-F. (2007). Design, synthesis, and aqueous aggregation behavior of nonionic single and multiple thermoresponsive polymers. Langmuir.

[52] De P., Gondi S.R., Sumerlin B.S. (2008). Folate-conjugated thermoresponsive block copolymers: Highly efficient conjugation and solution self-assembly. Biomacromolecules.

[53] Vogt A.P., Sumerlin B.S. (2008). Tuning the temperature response of branched poly (N-isopropylacrylamide) prepared by RAFT polymerization. Macromolecules.

[54] Li H., Bapat A.P., Li M., Sumerlin B.S. (2011). Protein conjugation of thermoresponsive amine-reactive polymers prepared by RAFT. Polym. Chem..

[55] De P., Sumerlin B.S. (2013). Precision control of temperature response by copolymerization of di (ethylene glycol) acrylate and an acrylamide comonomer. Macromol. Chem. Phys..

[56] Li M., Li H., De P., Sumerlin B.S. (2011). Thermoresponsive Block Copolymer–Protein Conjugates Prepared by Grafting-from via RAFT Polymerization. Macromol. Rapid Commun..

[57] Roy D., Cambre J.N., Sumerlin B.S. (2010). Future perspectives and recent advances in stimuli-responsive materials. Prog. Polym. Sci..

[58] Schattling P., Jochum F.D., Theato P. (2014). Multi-stimuli responsive polymers–the all-in-one talents. Polym. Chem..

[59] Wei M., Gao Y., Li X., Serpe M.J. (2017). Stimuli-responsive polymers and their applications. Polym. Chem..

[60] Kubo T., Easterling C.P., Olson R.A., Sumerlin B.S. (2018). Synthesis of multifunctional homopolymers via sequential post-polymerization reactions. Polym. Chem..

[61] Kale T.S., Klaikherd A., Popere B., Thayumanavan S. (2009). Supramolecular assemblies of amphiphilic homopolymers. Langmuir.

[62] Vasilevskaya V.V., Govorun E.N. (2019). Hollow and Vesicle Particles from Macromolecules with Amphiphilic Monomer Units. Polym. Rev..

[63] Zhang J., Liu K., Müllen K., Yin M. (2015). Self-assemblies of amphiphilic homopolymers: Synthesis, morphology studies and biomedical applications. Chem. Commun..

[64] Carothers W.H. (1931). Polymerization. Chem. Rev..

[65] Odian G. (1991). Principles of Polymer Chemistry.

