# Supplementary material for: Thermoresponsive Poly(ß-hydroxyl amine)s: Synthesis of a New Stimuli Responsive Amphiphilic Homopolymer Family through Amine-Epoxy ‘Click’ Polymerization
Source: Polymers (Basel). 2019 Nov 25;11(12):1941. doi: 10.3390/polym11121941 (PMC6961043; doi:10.3390/polym11121941)
Supplement: Supplementary file 1 [file polymers-11-01941-s001.pdf]

Supplementary Information

Thermoresponsive Poly(ß-hydroxyl amine)s: Synthesis of a New Stimuli Responsive Amphiphilic Homopolymer Family through Amine-Epoxy 'Click' Polymerization

Jeonghui Hong and Anzar Khan*

Email: anzar@korea.ac.kr

Experimental Details

General Methods and Materials:

Poly(ethylene glycol) diglycidyl ether (Average M_n = 500), methyl amine, ethyl amine, propyl amine, butyl amine, pentyl amine, hexyl amine, 2-methoxyethyl amine, 2-ethoxyethanamine, and 1,8-diaminooctane were purchased from Sigma Aldrich and used without any further purification. Gel permeation chromatography (GPC) was done in THF using a Waters system supplied with a refractive index detector (Water 2414 refractive 5 index detector, Waters 1515 pump). GPC measurements were conducted with three styragel HR 0.5, HR 2, and HR4 Columns. PS standards in THF were used for the relative molecular weight determinations. NMR spectra were measured on a Varian NMR System 500 MHz spectrometer, using $CDCl_3$ and DMSO as the deuterated solvents at 500 MHz for ^{1}H-NMR and 125 MHz for ^{13}C-NMR. ^{1}H-NMR spectra were measured on UNITY-INOVA 500 from VARIAN. INC., using D_2O at 500 MHz. The UV/Vis measurements were conducted on a Lambda 265 UV/Vis-spectrometer from Perkin-Elmer. Dynamic Light Scattering (DLS) was conducted using (Brookhaven Instruments Corporation) with a 636 nm wavelength laser beam at the angle ϑ of 90°. Fourier Transform infrared (FT-IR) spectrometer with ATR (attenuated total reflection) was conducted on Agilent Cary 630 FTIR.

Synthesis:

General procedure using example of polymer **3d**: Butyl amine (1.00 g, 13.6 mmol) was added very slowly and drop-wise to an ice-cooled solution of monomer **2** (6.80 g, 13.6 mmol) in water (1.95 mL). Both monomers were used as received from commercial suppliers without any further purification. After complete addition of the amine monomer, the ice-bath was removed and the reaction mixture was stirred

at room temperature for 48 hours. After this time, the reaction mixture was freeze-dried and used for further studies. **3d**: ^{1}H-NMR (500 MHz, Chloroform-d_3): δ 3.83 – 3.31 (s, 40H), 2.59 – 2.36 (m, 6H), 1.36 (t, J = 7.2 Hz, 2H), 1.27 – 1.16 (m, 2H), 0.83 (t, J = 7.3 Hz, 3H). IR (cm^{-1}): 3449, 2864, 1738, 1455, 1351, 1294, 1230, 1095, 864, 740, 540. GPC (THF): M_n = 6700, M_w = 8700, PDI (M_w/M_n) = 1.3. **3a**: ^{1}H NMR (500 MHz, Chloroform-d_3) δ 3.82 – 3.32 (m, 40H), 2.49 – 2.29 (m, 4H), 2.24 (d, J = 7.4 Hz, 3H). **3b**: ^{1}H NMR (500 MHz, Chloroform-d_3) δ 3.81 – 3.33 (m, 40H), 2.64 – 2.39 (m, 6H), 0.94 (t, J = 7.0, Hz, 3H). GPC (THF): M_n = 2900, M_w = 3600, PDI (M_w/M_n) = 1.2. **3c**: ^{1}H NMR (500 MHz, Chloroform-d) δ 3.82 – 3.31 (m, 40H), 2.45 (m, 6H), 1.45 – 1.32 (m, 2H), 0.79 (t, J = 7.4 Hz, 3H). GPC (THF): M_n = 4900, M_w = 7100, PDI (M_w/M_n) = 1.4. **3e**: ^{1}H NMR (500 MHz, Chloroform-d) δ 3.83 – 3.31 (m, 40H), 2.58 – 2.36 (m, 6H), 1.36 (m, 2H), 1.29 – 1.12 (m, 4H), 0.81 (t, J = 7.1 Hz, 3H). GPC (THF): M_n = 5500, M_w = 7900, PDI (M_w/M_n) = 1.4. **3f**: ^{1}H NMR (500 MHz, Chloroform-d) δ 3.82 – 3.32 (m, 40H), 2.56 – 2.35 (m, 6H), 1.35 (m, 2H), 1.20 (m, 6H), 0.80 (t, J = 6.8 Hz, 3H). GPC (THF): M_n = 6000, M_w = 9500, PDI (M_w/M_n) = 1.5. **3g**: ^{1}H NMR (500 MHz, Chloroform-d) δ 3.77 – 3.30 (m, 40H), 3.25 (s, 3H), 2.80 – 2.43 (m, 6H). GPC (THF): M_n = 4500, M_w = 7100, PDI (M_w/M_n) = 1.5. **3h**: ^{1}H NMR (500 MHz, Chloroform-d) δ 3.83 – 3.31 (m, 40H), 2.83 – 2.44 (m, 6H), 1.11 (t, J = 7.0 Hz, 3H). GPC (THF): M_n = 4600, M_w = 7000, PDI (M_w/M_n) = 1.5.

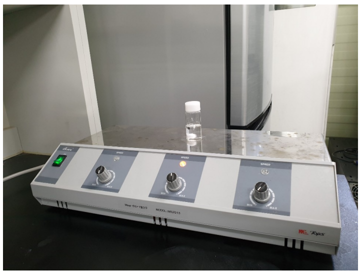

Figure S1. A (multi)gram scale synthesis can be carried out in a screw-cap glass vial using magnetic stir bars.

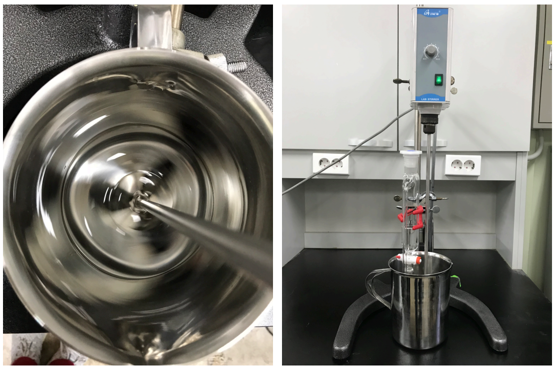

Figure S2. Large-scale synthesis (512 grams in this digital picture) requires mechanical stirring due to a highly viscous nature of the polymerization mixture. At large scale, a very slow addition of amine is recommended (1 drop/minute) and the aqueous reaction mixture must be ice-cooled. The outlet of amine reservoir is recommended to be as close as possible to the surface of the polymerization mixture. This is because as soon as the amine becomes part of the viscous aqueous solution of PEG-monomer it does not leave the system. A syringe-pump can also be used to control the addition of amine. The plastic cannula from the syringe (due to its small surface area) can be dipped into the polymerization mixture without disturbing the stirring. In this case, the amine smell and evaporation can be avoided. The ice bath can be removed after complete amine addition and the polymerization reaction can be allowed to proceed at room temperature as shown in the digital picture here.

Study of thermoresponsive behavior:

Lambda 265 UV/Vis-spectrometer from Perkin-Elmer was used for transmittance measurements at 600 nm by heating the aqueous solution by 0.5 °C with 10 mg/mL polymer concentration. The sample temperature was measured with temperature probe (thermometer equipped with Probe k-type thermocouple (-160/+700°C, Φ1.0×300mm, AS1.5522.04 from AS ONE) in cuvette at each interval. The equilibration time at each temperature was 10 minutes. The coil to globule phase transition is given by the temperature of the inflection point of the heating curve in this work.

Dynamic light scattering (DLS) measurement:

Dynamic light scattering measurements were done by using (Brookhaven Instruments Corporation) with a 636 nm wavelength laser beam. The scattering

angle is 90°. CONTIN method was used for autocorrelation function analyses. D_{mean} is the mean size of the intensity distribution. DLS experiments were conducted with a heating and cooling cycle by increasing and decreasing temperature by 2 °C for each step. A polymer concentration of 10 mg/mL was used for each measurement. An equilibration time of 10 minutes was used.

Swelling/Deswelling experiments:

Monomer **2** (3.47 g), crosslinker octyl diamine (0.1 g, 10 mol%), and butyl amine (0.41 g) were mixed in water (0.99 g) and allowed to crosslink at room temperature for 24 hours and then freeze-dried. The mass of the freeze-dried gel sample was measured. The sample was swelled in deionized water for 80 hr and then the swollen sample was deswelled at 40 °C in deionized water for 12 hr. During swelling and deswelling procedure, after water was removed from the surface of gel with filter paper, the mass of swollen gel was measured at each time intervals. Swelling ratio was calculated by the equation [Swelling ratio = $\{(W_S-W_d)/W_d\} \times 100$], W_S is the mass of swollen gel, W_d is the mass of the freeze-dried gel.

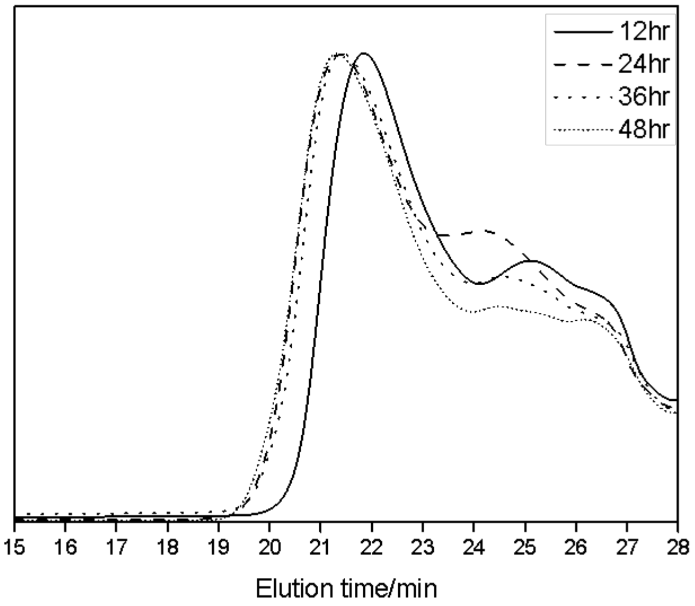

Figure S3. Crude gel permeation chromatography (GPC) traces during the polymerization of **3d** in a 50 wt.% aqueous solution.

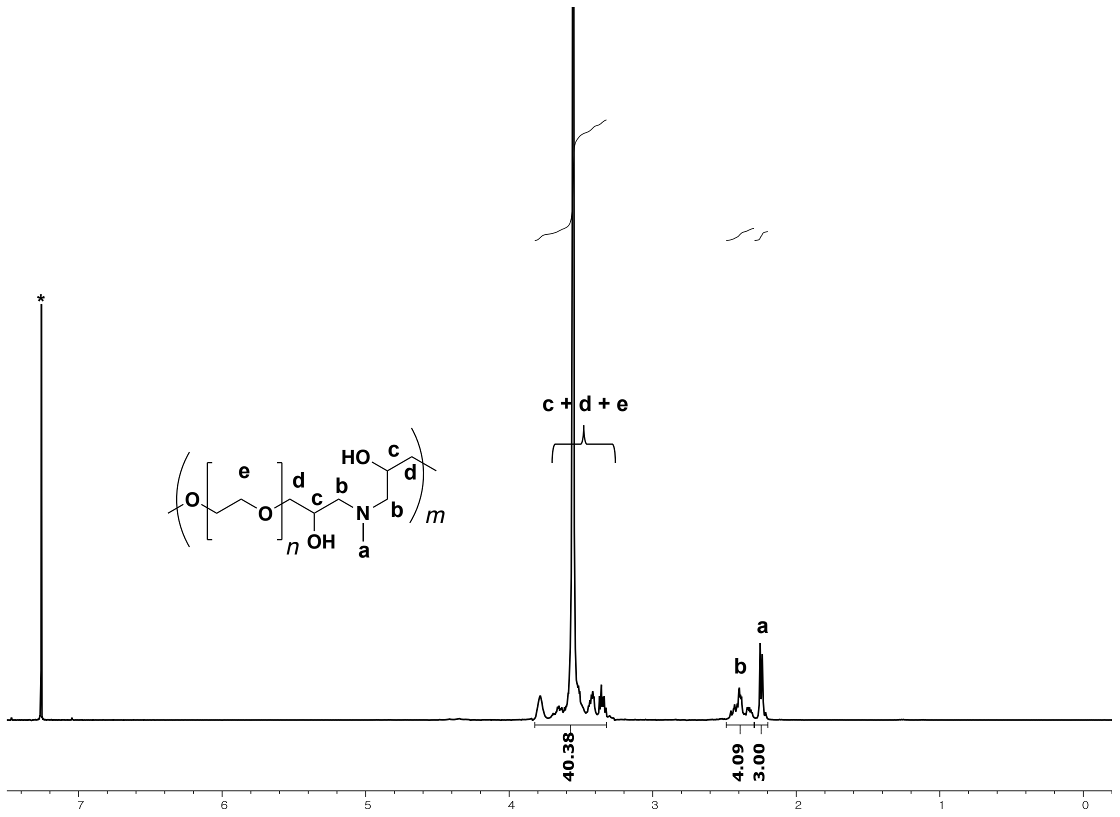

Figure S4. ^{1}H-NMR of **3a** in deuterated chloroform.

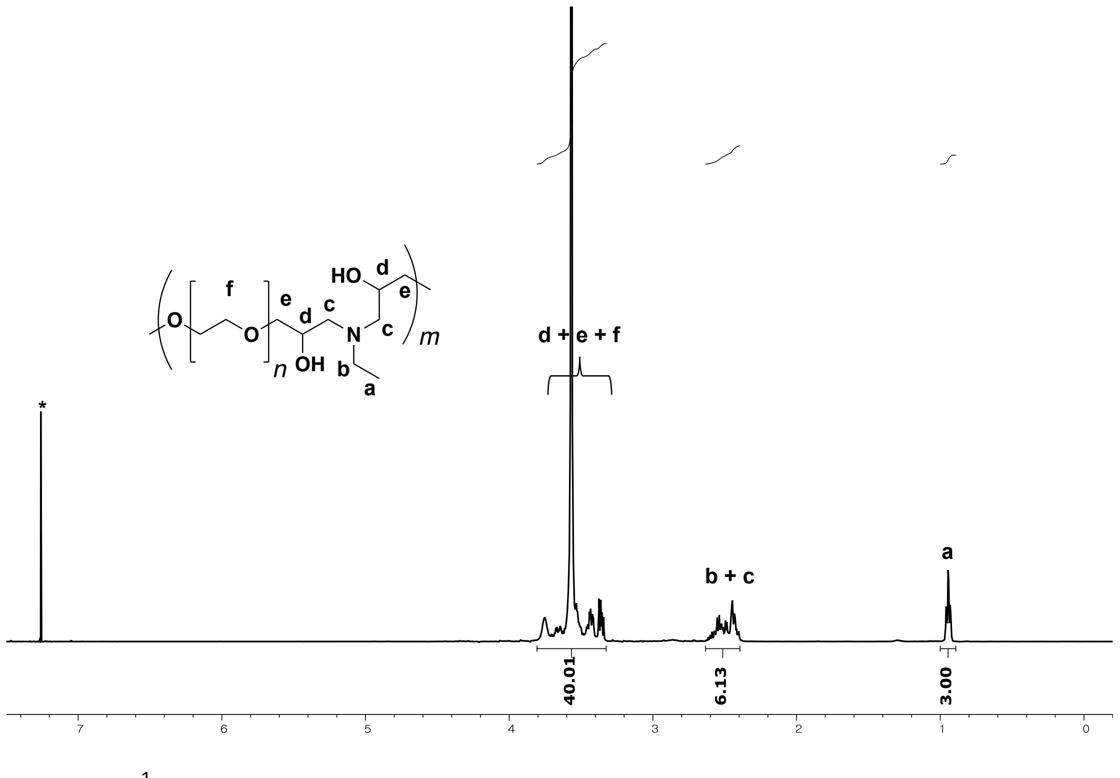

Figure S5. ^{1}H-NMR of **3b** in deuterated chloroform.

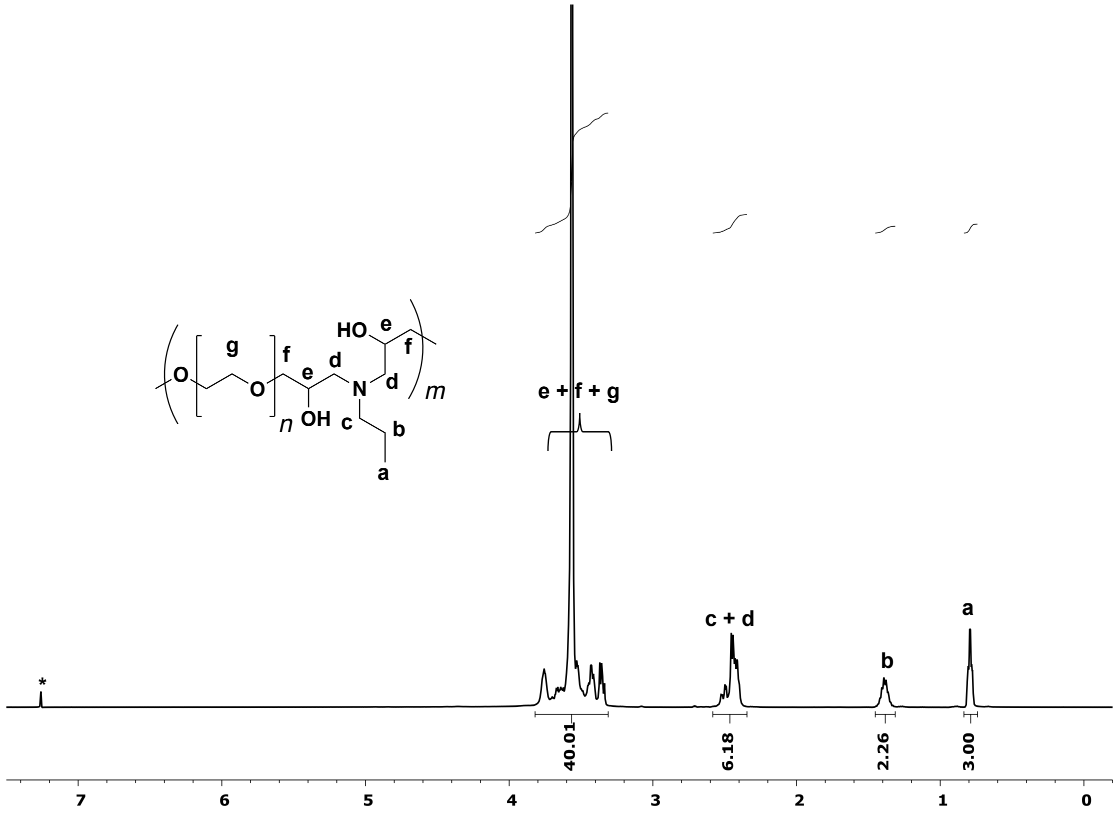

Figure S6. ^{1}H-NMR of **3c** in deuterated chloroform.

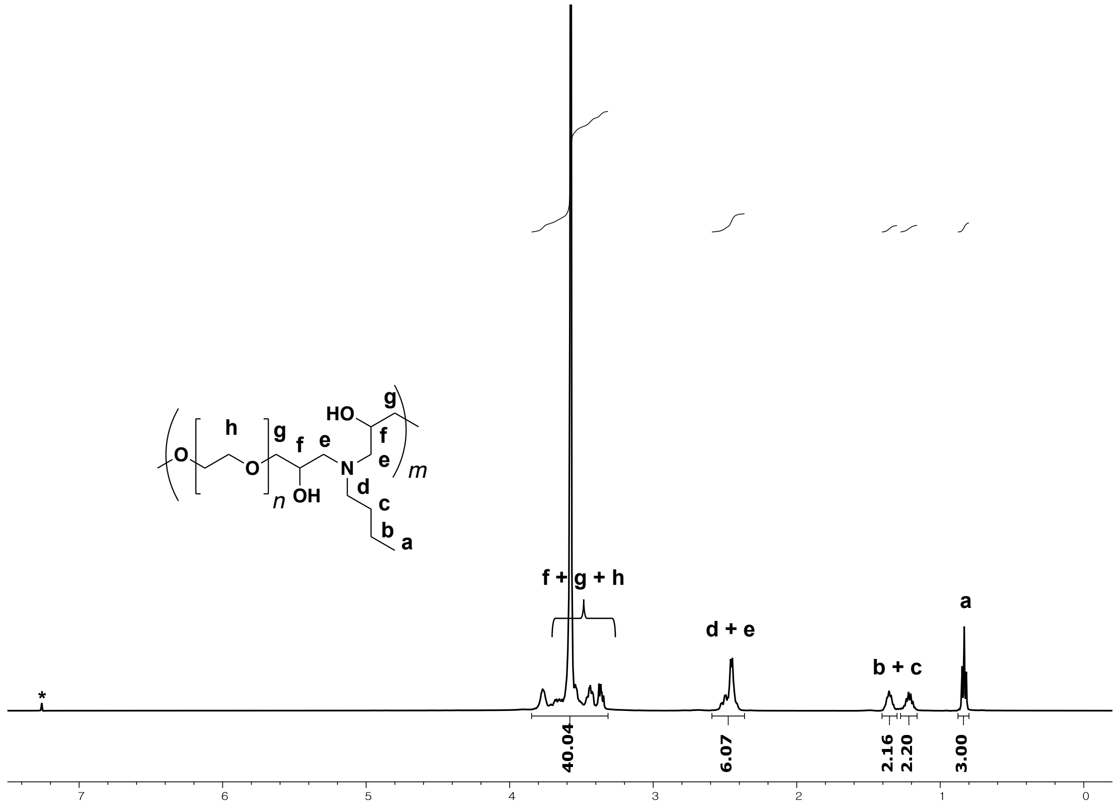

Figure S7. ^{1}H-NMR of **3d** in deuterated chloroform.

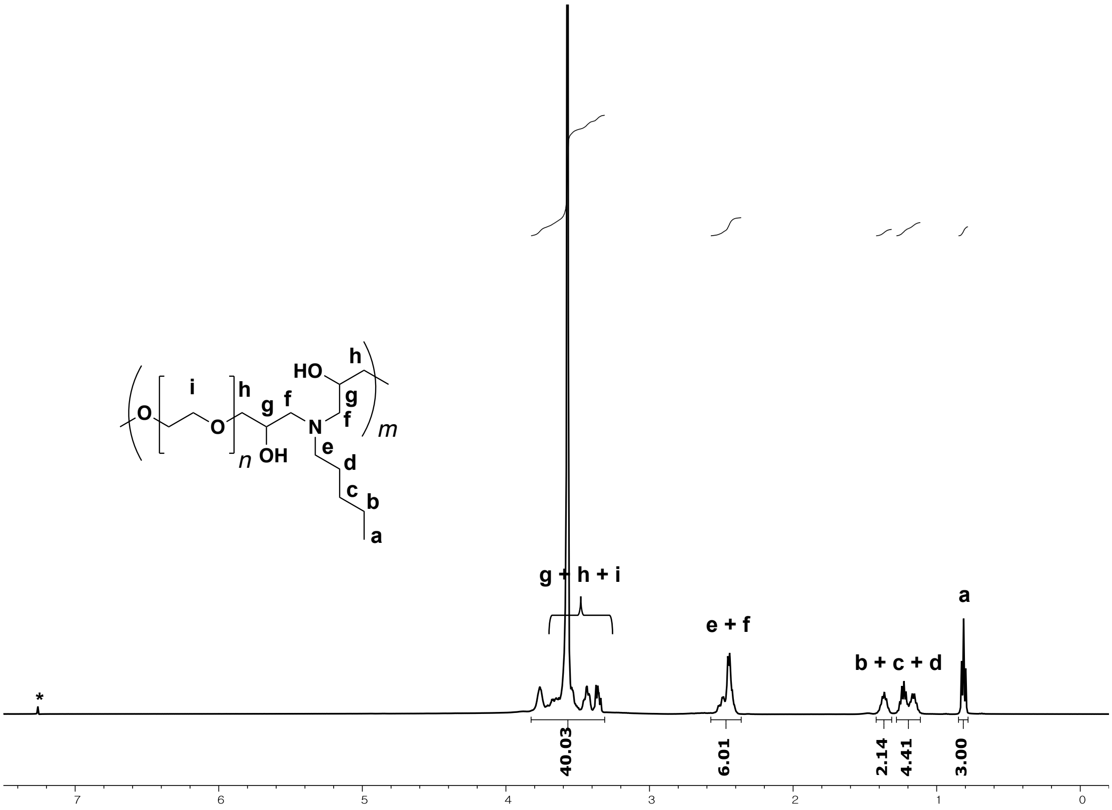

Figure S8. ^{1}H-NMR of **3e** in deuterated chloroform.

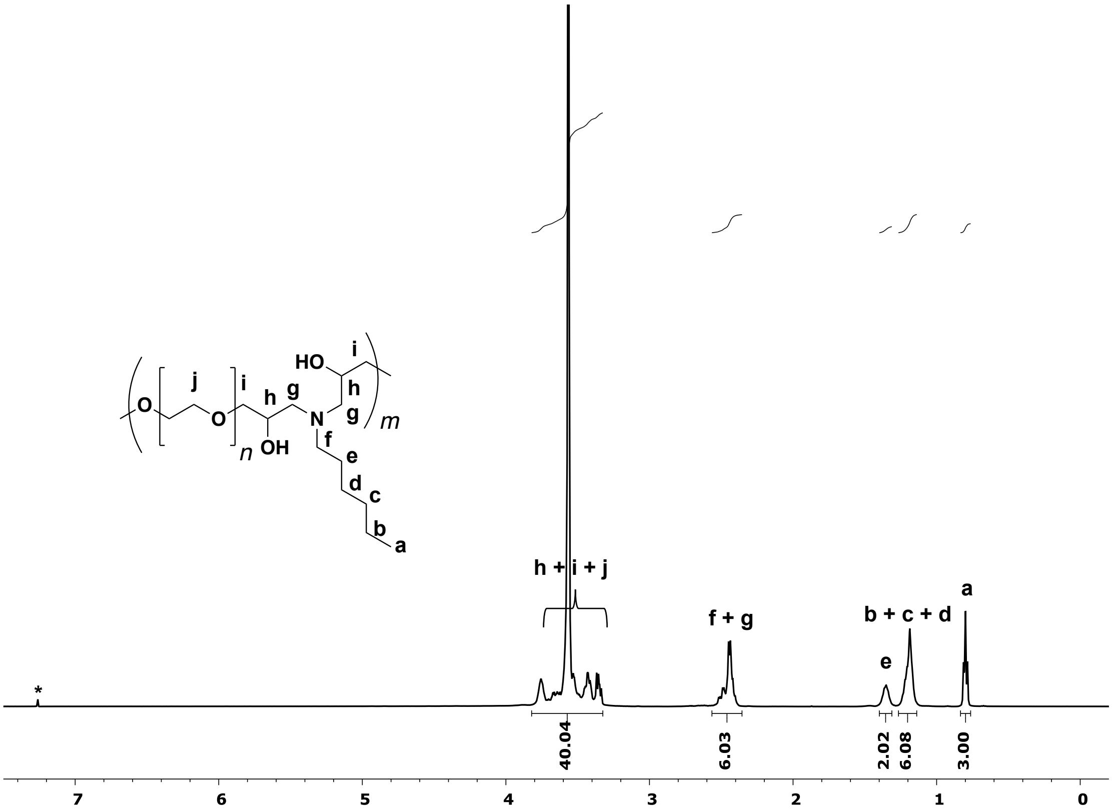

Figure S9. ^{1}H-NMR of **3f** in deuterated chloroform.

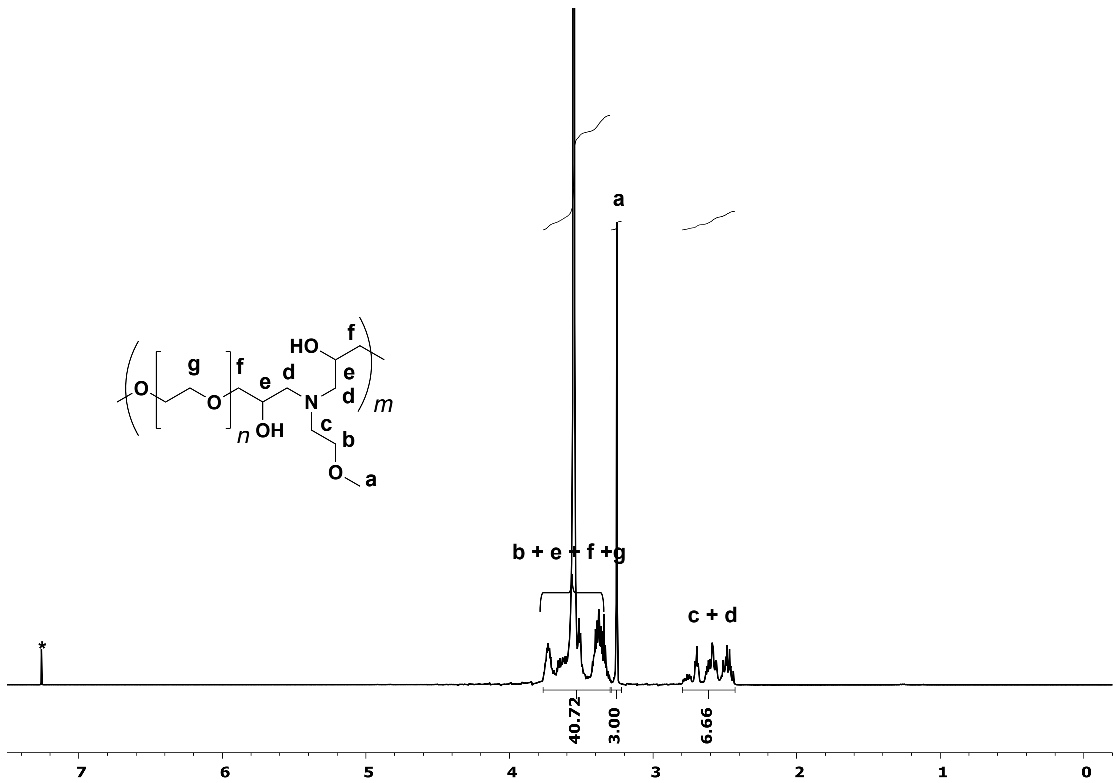

Figure S10. ^{1}H-NMR of **3g** in deuterated chloroform.

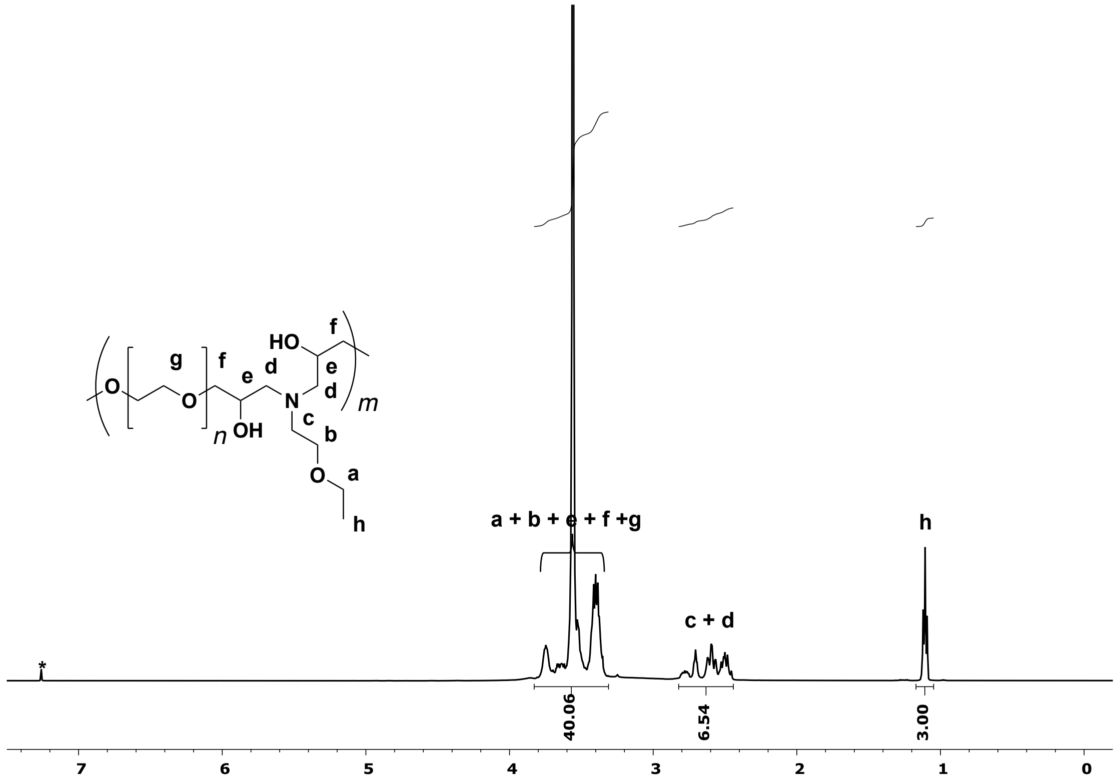

Figure S11. ^{1}H-NMR of **3h** in deuterated chloroform.

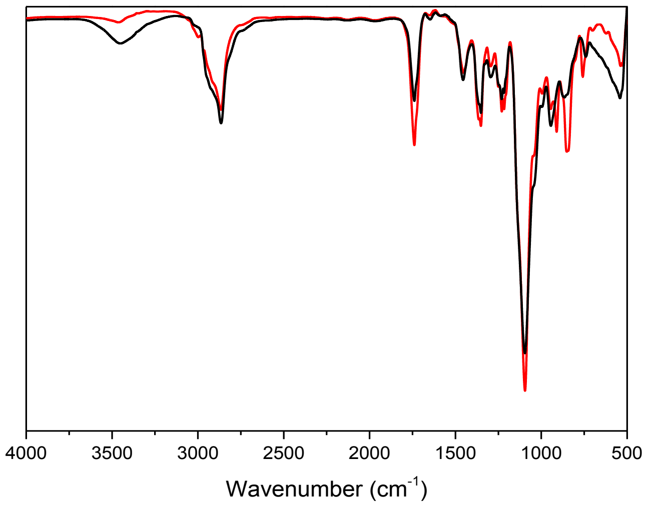

Figure S12. IR spectra of monomer **2** (red) and polymer **3** (black).

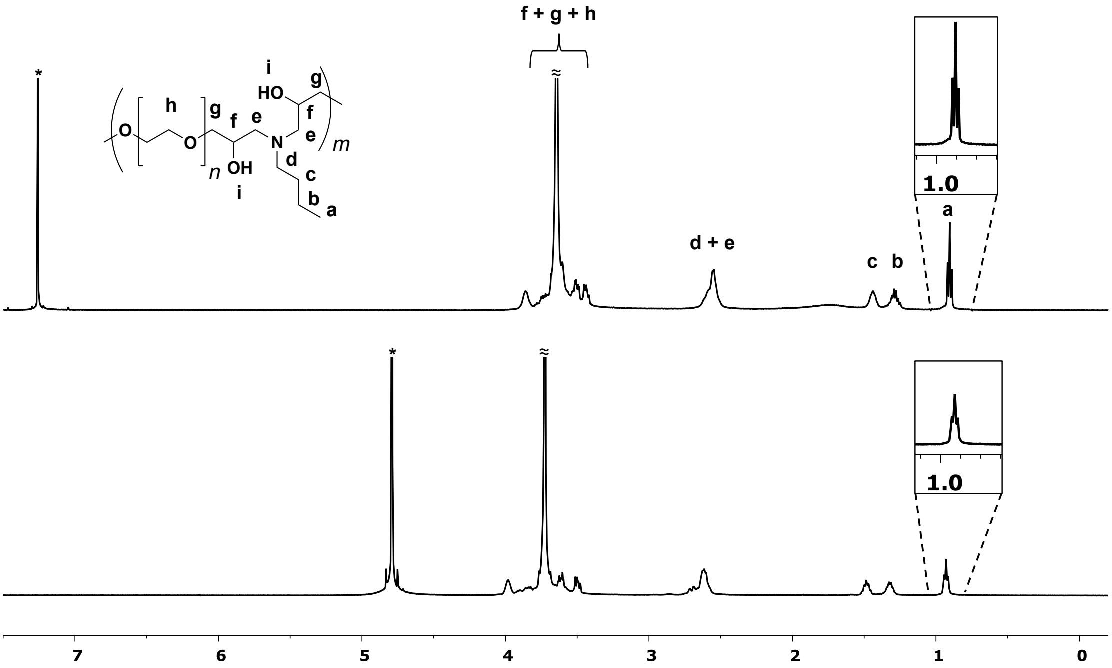

Figure S13. ^{1}H-NMR of **3d** in deuterated chloroform (top) and deuterated water (bottom).

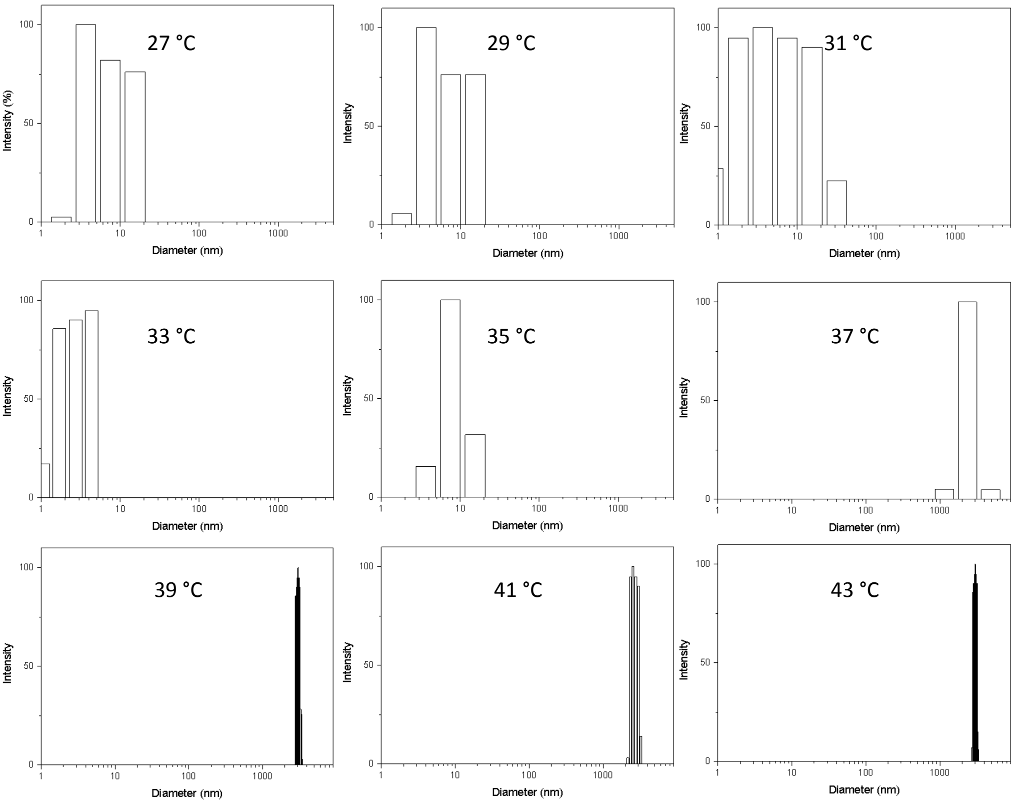

Figure S14. DLS of an aqueous solution of polymer **3d** before and after the inflection point temperature of 36 °C.

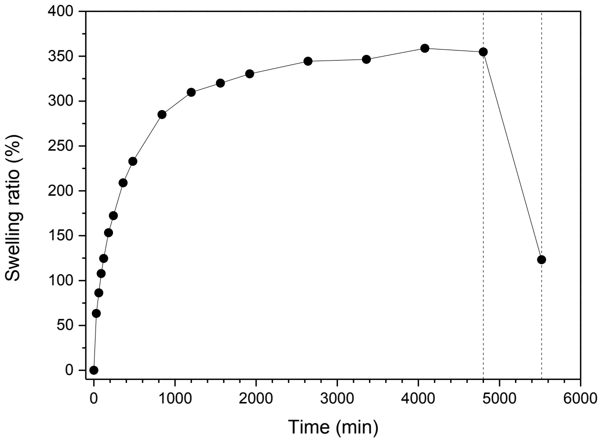

Figure S15. Evolution of hydrogel swelling as a function of time. At t = 4800 min, the temperature of the aqueous medium was raised from room temperature to 40 °C.
